# Water stabilization of Zr_6_-based metal–organic frameworks *via* solvent-assisted ligand incorporation†
†Electronic supplementary information (ESI) available. See DOI: 10.1039/c5sc01784j


**DOI:** 10.1039/c5sc01784j

**Published:** 2015-07-01

**Authors:** Pravas Deria, Yongchul G. Chung, Randall Q. Snurr, Joseph T. Hupp, Omar K. Farha

**Affiliations:** a Departments of Chemistry and Chemical and Biological Engineering , Northwestern University , 2145 Sheridan Road , Evanston , Illinois 60208 , USA . Email: j-hupp@northwestern.edu ; Email: o-farha@northwestern.edu; b Department of Chemistry , Faculty of Science , King Abdulaziz University , Jeddah , Saudi Arabia

## Abstract

Water stability in metal–organic frameworks (MOFs) is critical for several practical applications. While water instability is mainly thought to stem from linker hydrolysis, MOFs with strong, hydrolysis-resistant metal-linker bonds can be susceptible to damage by capillary forces, which cause cavities and channels to collapse during activation from water. This study utilizes metal node functionalization as a strategy to create vapor-stable and recyclable MOFs.

## Abstract 2

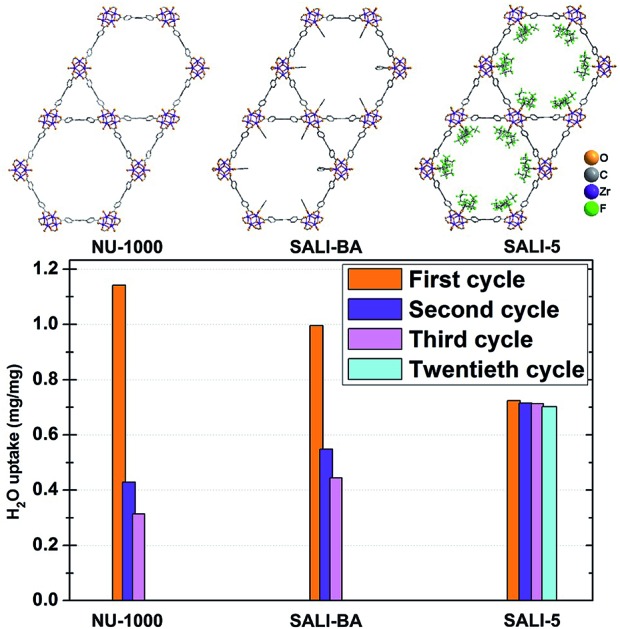
Water stability in metal–organic frameworks (MOFs) is critical for several practical applications; we report here fundamental understanding how capillary forces induce damage to MOFs and highlight that metal node functionalization as a strategy to create vapor-stable and recyclable MOFs.

## Introduction

Porous metal–organic frameworks (MOFs) are hybrid solid-state compounds^1^ that have attracted voluminous research efforts for applications relevant to gas storage and separation,^2^ chemical catalysis,^3^ and optoelectronic^4^ and thermoelectric devices.^5^ However, many of these applications require recyclability, which is intimately tied to the stability of the corresponding frameworks in ambient conditions as well as in the presence of water.^5^,^6^ It is worth noting that only a modest fraction of the total known MOFs are both thermally and chemically stable. Stable MOFs include those derived from nodes with high valent metal ions^7^ and some imidazolate, pyrazolate, and multitopic carboxylate linkers. Among the more remarkable examples are ZIFs,^8^ pyrazolate-based frameworks,^9^ MILs,^10^ and Zr^IV^-based UiOs.^7^,^11^ While the microporous UiO-66 framework, consisting of 12-connected [Zr_6_(μ_3_–O)_4_(μ_3_–OH)_4_]^12+^ nodes linked through 1,4-benzene-dicarboxylates (BDC),^7^ was established to be mechanically,^7^,^12^ thermally,^7^ hydrolytically and chemically stable,^7^,^13^ other Zr_6_ based MOFs with larger pores, such as UiO-67, constructed with a longer linker (BPDC, 4,4′-biphenyl-dicarboxylate), have often been found to lose porosity upon exposure and activation from water.13b,^14^ We recently showed that the instability of these Zr_6_-based MOFs is not due to hydrolysis, which is an energetically disfavored process (Δ*G* ≈ +26 kcal mol^–1^ with Δ*G*‡ ≈ +38 kcal mol^–1^) owing to a strong Zr^IV^-carboxylate connectivity, but instead stems from capillary-force-driven channel collapse during removal of water.13*a* The water-exposed MOF sample, when exchanged with a solvent exerting less capillary force, was shown to retain permanent porosity by thermal activation under dynamic vacuum.13a,^15^ Some applications of MOFs, however, may not be compatible with stepwise solvent evacuation.^6^,13b,^16^ The availability of an alternative stabilization method clearly would be desirable.

**Fig. 1:**
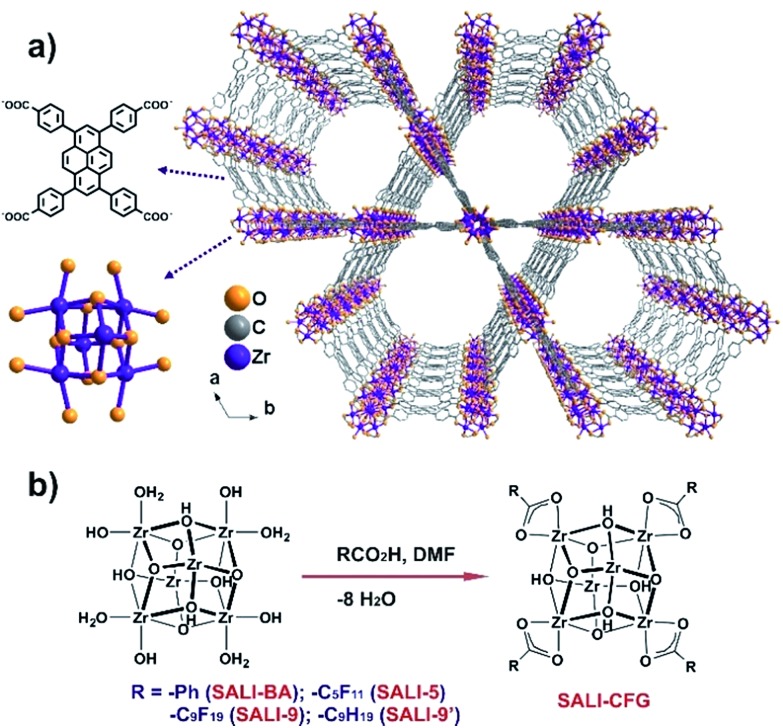
(a) Structure of **NU-1000** (H-atoms were removed for clarity) and (b) preparation of **SALI-CFG**s *via* Solvent-Assisted Ligand Incorporation (SALI).

Given the breadth of applications specifically of Zr_6_-based MOFs to condensed-phase chemistry,^17^ we have focused on understanding the origin of such capillary-force-driven collapse, while devising a strategy that would enable MOFs to be recycled (*i.e.* repetitively solvent evacuated) *via* simple thermal activation. The Zr_6_-based mesoporous MOFs are intriguing, particularly those featuring eight-connected [Zr_6_(μ_3_–O)_4_(μ_3_–OH)_4_(–OH)_4_(–OH_2_)_4_]^8+^ nodes linked by tetra-carboxylate ligands (Fig. 1a) giving rise to framework structures with hydroxyl and aqua ligands^18^ protruding into mesoporous channels. These non-bridging hydroxyl and aqua ligands provide polar and tunable primary interaction points or ‘anchors’ for water clusters to attach^5^ and are, thus, ideal for studying the role of polar nodes in capillary-force-driven pore collapse.

We have established that the non-bridging hydroxyl and aqua ligands of the Zr_6_-nodes of the mesoporous MOF **NU-1000** [molecular formula Zr_6_(μ_3_–O)_4_(μ_3_–OH)_4_(–OH)_4_(–OH_2_)_4_(TBAPy)_2_; Fig. 1a; H_4_TBAPy is the 1,3,6,8-tetrakis(*p*-benzoic-acid)pyrene]17c,^18^-^19^ can facilitate a variety of node-functionalization schemes including metalation *via* AIM (**A**tomic layer deposition **I**n a **M**etal–organic framework)17*c* and grafting organic chemical functionalities *via* SALI (**S**olvent-**A**ssisted **L**igand **I**ncorporation; Fig. 1b).^19^,^20^ These modifications not only facilitate various chemical processes,^19^,^20^ but also potentially provide a means to control the pore diameter and the accessibility of the nodes by condensed water. For example, a SALI-derived **NU-1000** framework possesses a node coordination similar to that established in the UiO-66 structure,^19^*i.e.* a [Zr_6_(μ_3_–O)_4_(μ_3_–OH)_4_]^12+^ node with twelve carboxylate ligands without any terminal hydroxyl or aqua ligand (Fig. 1b). Recent literature reports have established that in microporous UiO-66 and isostructural MOF-801 (with fumarate as the linker),^5^,^6^ the bridging hydroxo ligands on the Zr_6_-oxo node play a key role as a primary interaction site for the guest water molecules, which form a hydrogen bonding network that facilitates further uptake of water molecules. Since SALI-decoration in **NU-1000** replaces the terminal hydroxo and aqua ligands at the Zr_6_-oxo nodes, the incoming carboxylate functional groups (CFGs) of various size not only reduce the pore volume but also potentially produce a ‘protected’ node that interacts less strongly with water molecules (Fig. 1b).^5^,^6^,^21^

## Results and discussion

**Fig. 2:**
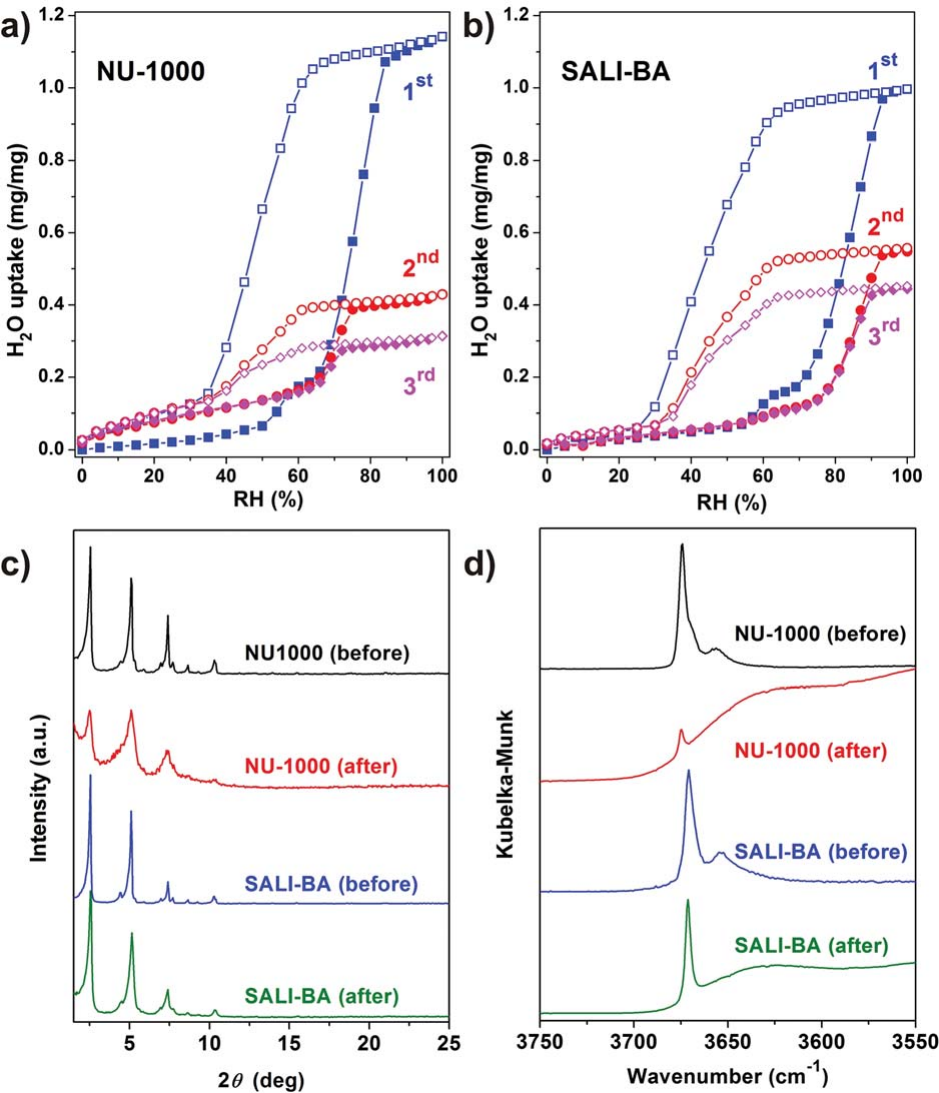
Multi-cycle water vapor adsorption (solid symbols) and desorption (open symbols) isotherms of (a) **NU-1000** and (b) **SALI-BA** at 298 K; (c) PXRD patterns and (d) DRIFTS data for **NU-1000** and **SALI-BA** samples before and after three water isotherm cycles.

**Fig. 3:**
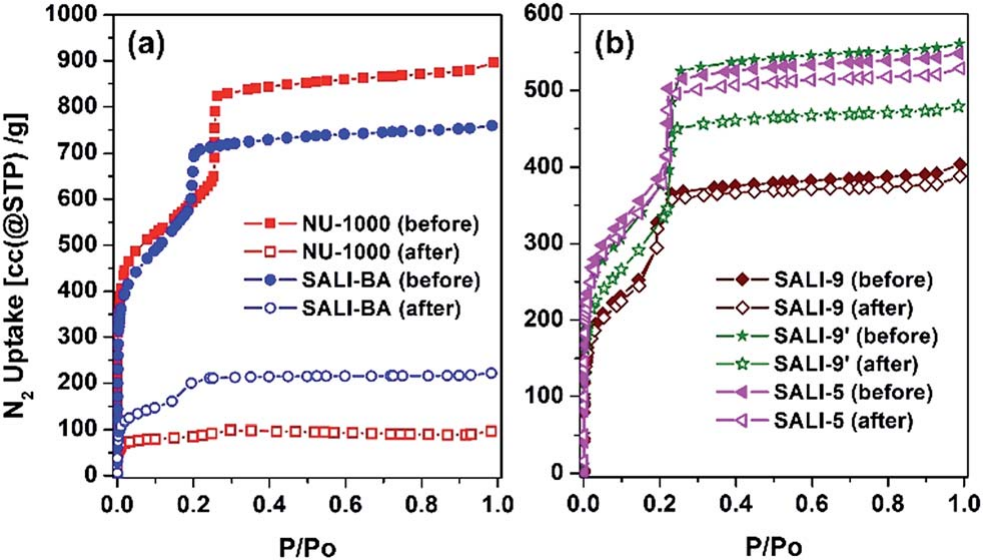
N_2_ adsorption isotherms for (a) **NU-1000** and (b) **SALI-CFG** samples before (solid symbol) and after (open symbol) multi-cycle (twenty cycles, except for three-cycle experiments for **NU-1000** and **SALI-BA**) water vapor sorption experiments.

Given that **NU-1000** undergoes capillary-force-driven pore collapse when activated (thermal evacuation) from its liquid-water-filled form,13*a* we wanted to test its stability under water vapor conditions at various vapor partial pressures (or relative humidities, RH) using N_2_ as carrier gas at a constant total pressure of 1 bar.^22^ As can be seen in Fig. 2a, at RH ≈ 70%, the mesoporous **NU-1000** channels undergo capillary condensation and reach saturation with 1.14 mg mg^–1^ total uptake (corresponding to a water accessible pore volume of 1.14 cc g^–1^, where the N_2_ accessible pore volume = 1.4 cc g^–1^).^23^ Removal of the condensed water, during the desorption cycle, was achieved by progressive lowering of the vapor concentration or partial pressure with dry N_2_ carrier gas and occurs with considerable hysteresis. However, the second adsorption isotherm, under identical conditions, evinces only a 0.43 mg mg^–1^ saturation uptake. The decrease in the total uptake indicates a decrease in the porosity, which possibly commenced during the first desorption cycle. Likewise, the third adsorption shows 0.31 mg mg^–1^ of total uptake with a further decrease of the porosity. The powder X-ray diffraction (PXRD) pattern of the recovered sample revealed a lower crystallinity (Fig. 2c), as the diffraction peaks centered at 2.55, 5.1 and 7.4 degrees (2*θ*) are significantly broadened and the peaks at higher angles less intense. Consistent with these findings, the N_2_ isotherm of the recovered (after the third cycle), thermally activated sample shows significant reductions of both the BET surface area (320 m^2^ g^–1^) and the N_2_ accessible total pore volume down (0.15 cc g^–1^) (Fig. 3a and Table 1).

**Table 1:** BET surface areas, pore volumes, multi-cycle water uptake for **NU-1000** and **SALI-CFG** Samples

MOF	Ligand	BET surface area (m^2^ g^–1^)	Pore volume (cc g^–1^)	Multi-cycle saturation H_2_O uptake (mg mg^–1^); *T* = 298 K				BET surface area (m^2^ g^–1^) after H_2_O sorption
				1^st^	2^nd^	3^rd^	20^th^	
**NU-1000**	OH^–^, H_2_O	2145	1.46	1.14	0.43	0.31	—	320
**SALI-BA**	C_6_H_5_CO_2_^–^	2005	1.21	1.00	0.56	0.45	—	570
**SALI-5**	CF_3_(CF_2_)_4_CO_2_^–^	1270	0.85	0.72	0.72	0.71	0.70	1250
**SALI-9**	CF_3_(CF_2_)_8_CO_2_^–^	900	0.63	0.44	0.43	0.43	0.43	890
**SALI-9′**	CH_3_(CH_2_)_8_CO_2_^–^	1190	0.87	0.78	0.73	0.72	0.68	1170

**Fig. 4:**
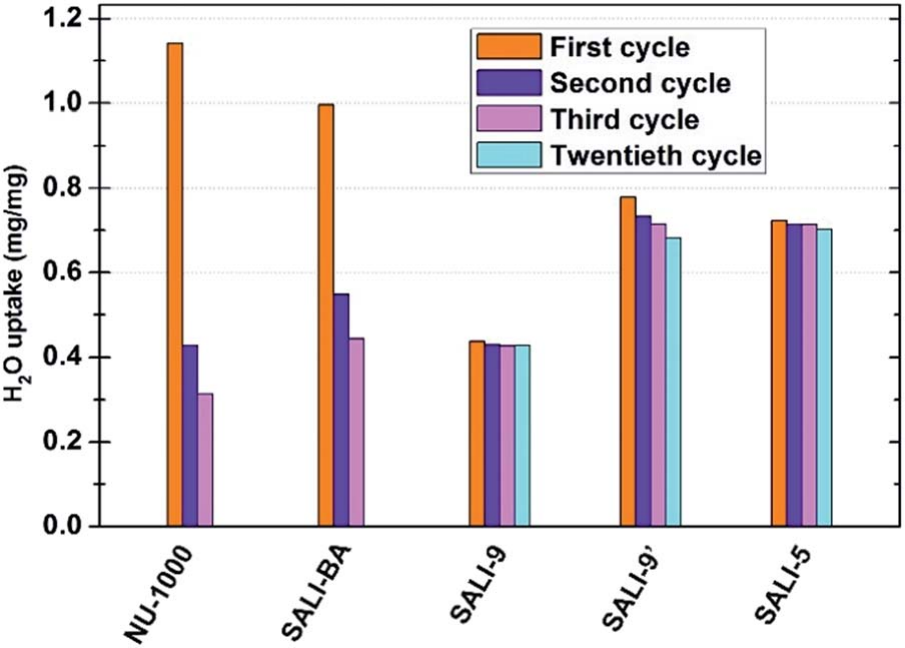
Saturation water uptake for **NU-1000** and **SALI-CFG** samples in multi-cycle water vapor sorption experiments recorded at 298 K.

To gain insight into the role of the polar hydroxyl and aqua ligands, we compared the results for **NU-1000** with those obtained benzoate functionalized **NU-1000** (termed **SALI-BA**), where the accessible terminal hydroxyl and aqua ligands are replaced by benzoate ligands (Fig. 1b). Similar to **NU-1000**, **SALI-BA** is subject to capillary condensation at RH ≈ 70% and reaches a water saturation uptake of 1.0 mg mg^–1^, which tracks with the N_2_ accessible pore volume (1.21 cc g^–1^; Fig. 2b; Table 1). **SALI-BA** exhibited a diminished uptake during the second adsorption. However, the corresponding reduction of the pore volume is less severe (∼44%) than that observed for the unfunctionalized **NU-1000** (∼62%). The magnitude of the pore volume reduction suggests that a benzoate functionalized node (1) provides a less effective ‘anchoring’ site and (2) engenders lower pore volume for the capillary-condensed water, rendering a lower degree of damage during the desorption. The corresponding PXRD pattern and the N_2_ isotherm of the recovered (after third cycle), thermally activated sample indicated better retention of crystallinity and porosity relative to the unfunctionalized compound (see Fig. 2c and 3a). DRIFTS data of the recovered **SALI-BA** sample show retention of the characteristic bridging hydroxyl peak at 3671 cm^–1^ whereas the recovered unfunctionalized **NU-1000** displays a broad brand at the expense of the sharp peaks at 3674 and 3671 cm^–1^ associated with the terminal and bridging hydroxyl and the terminal aqua ligands, respectively (Fig. 2d). These data suggest node structure alteration and possibly a partially pore-collapsed framework. We now turn our attention to SALI-CFG materials featuring flexible functional groups that may protect the node further by inhibiting access of condensed water to the MOF node. Perfluorodecanoic-acid-functionalized **NU-1000** (termed **SALI-9**)19*b* is an example of a highly-stable mesoporous MOF with a reduced pore volume, rendering a total water vapor uptake that is ∼40% as great as that of unfunctionalized **NU-1000** (Fig. 4, ESI-4a;† Table 1). Remarkably, over the course of 20 cycles of water vapor sorption/desorption, the porosity and crystallinity of **SALI-9** remain essentially unchanged. It is worth noting that the N_2_-accessible pore volume of **SALI-9** sample is measured to be 0.63 cc g^–1^,19*b**i.e.* somewhat lower than that of the parent material and consistent with the volumetric demands of the perfluoroalkane chains themselves. Even though functionalization significantly reduces the MOF pore volume, it is interesting to note that water condensation,^5^ in **SALI-9**, commences at essentially the same vapor pressure as for **NU-1000** (Fig. 2). Corresponding contact angle measurements (Fig. ESI-7†), a bulk measurement of external-surface interactions with liquid water, indicate enhanced hydrophobicity for **SALI-9** relative to unfunctionalized **NU-1000**. The recovered sample (after 20 cycles) after thermal activation reveals essentially no degradation of the crystallinity, porosity and Zr_6_-oxo node structure (see Fig. 3, ESI-5 and 6†).

To understand if the acquired stability is due mainly to the hydrophobic nature of the perfluoroalkane decoration or to concurrent reduction of the pore size and, therefore, water cluster size, we studied the corresponding –C_9_H_19_ and the shorter –C_5_F_11_ chains in **NU-1000** (**SALI-9′** and **SALI-5**, respectively). Due to its lower molar mass compared to the corresponding –C_9_F_19_ variant, **SALI-9′** shows higher gravimetric vapor uptake (*ca.* 68% of that of the unfunctionalize MOF; Fig. 4, ESI-4†). The stability of **SALI-9′** to repetitive infilling and removal of water is nearly as good as that of **SALI-9**, as can be seen from a modest 10% decrease in saturation water uptake after 20 cycles of vapor sorption and desorption.^24^ The N_2_ isotherm, PXRD, and DRIFTS data (Fig. 3 and ESI 5 and 6†) of the recovered thermally activated sample reveal essentially complete retention of the crystallinity, porosity, and node coordination environment. Likewise, the shorter perfluoroalkane variant, **SALI-5**, shows excellent performance: significantly higher water uptake (∼63% of **NU-1000**) along with stability (Fig. 3 and 4; Table 1) similar to that of **SALI-9**.

Given that the pyrene macrocycle of the **NU-1000** linker is hydrophobic, these experimental results led us to hypothesize that within the mesoporous MOF channel, the polar [Zr_6_(μ_3_–O)_4_(μ_3_–OH)_4_(–OH)_4_(–OH_2_)_4_(–COO_8_)] node strongly interacts with the condensed water. Thus during the desorption cycle, condensed water can exert significant capillary force, which in turn results in pore collapse.13a,^15^ In the **SALI-CFG** samples, the terminal hydroxyl and aqua ligands were replaced with aromatic or alkyl carboxylates that limit the accessibility of the polar Zr_6_-oxo nodes by water. Effective protection is tied to both the size and hydrophobicity of the CFG introduced by the SALI reaction.

To gain molecular insight into this hypothesis, we performed molecular dynamics (MD) simulations for water in **NU-1000**, **SALI-BA** and **SALI-5**. (see ESI section 7 for details.†) The simulations predict that the number of water molecules sited within 15 Å of the Zr_6_-nodes varies with MOF identity in the order **NU-1000** > **SALI-BA** > **SALI-5** (Table S4†). Likewise the density of water in the hexagonal channels (which could affect the capillary force) is ranked as **NU-1000** > **SALI-BA** ∼ **SALI-5** (Table S3†). These results support our hypothesis that the improved stability in SALI-derived system is due to limited accessibility of water molecules near the Zr_6_-nodes.

## Conclusions

In conclusion, our results suggest that strong interactions of condensed water with the polar nodes within the framework of **NU-1000** can exert strain during removal of the water from the pores. While replacement of the polar hydroxyl and aqua ligands with aprotic, non-polar organic carboxylates can reduce the strength of the interaction of water with the node, a flexible alkyl chain can further limit the accessibility by water vapor. MD simulations revealed that a longer and flexible carboxylate-based ligand indeed provides more limited access of water molecules to the Zr_6_ nodes. Node functionalization, by methods such as SALI, can be an effective strategy for engendering MOF stability toward water removal and, therefore, MOF recyclability from liquid water without loss of permanent porosity.

## Supplementary Material

Supplementary information
