# Supplementary material for: Water stabilization of Zr6-based metal–organic frameworks via solvent-assisted ligand incorporation
Source: Chem Sci. 2015 Jul 1;6(9):5172–6. doi: 10.1039/c5sc01784j (PMC6088556; doi:10.1039/c5sc01784j)
Supplement: Supplementary file 1 [file SC-006-C5SC01784J-s001.pdf]

Electronic Supplementary Material (ESI) for Chemical Science.
This journal is © The Royal Society of Chemistry 2015

Supporting Information

Water Stabilization of Zr_6-Based Metal-Organic Frameworks via Solvent-Assisted Ligand Incorporation

Pravas Deria,[a] *Yongchul G. Chung,*[a] *Randall Q. Snurr,*[a] *Joseph T. Hupp,*[*, a]

and Omar K. Farha,[*, a, b]

[a] Departments of Chemistry and Chemical and Biological Engineering, Northwestern University, 2145 Sheridan Road, Evanston, Illinois 60208, United States.

[b] Department of Chemistry, Faculty of Science, King Abdulaziz University, Jeddah, Saudi Arabia.

Table of Contents

Contents	Page Number
S1. Materials	2
S2. Instrumentation	2
S3. Synthesis and characterization of SALI-derived materials	3
S4. Multi-cycle water vapor isotherms	4
S5. Characterization data for **NU-1000** and **SALI**-derived materials before and after multi-cycle water isotherms	4-5
S6. Water contact angle measurement	6
S7 Details of molecular dynamics (MD) simulation	6-9

S1. Materials

Solvents, acetone, *N,N*-dimethylformamide (DMF), and dichloromethane were purchased from Macron; deuterated dimethyl sulfoxide (d_6-DMSO) (Cambridge Isotopes, 99%), and deuterated sulfuric acid (Cambridge Isotopes, 96-98% solution in D_2O) were used as received without further purification. Decanonic acid was purchased from Aldrich. H_4TBAPy [1,3,6,8-tetrakis(p-benzoic-acid)pyrene][1] was synthesized according to previous procedure.[2] Microcrystalline **NU-1000** was prepared solvothermally according to a published procedure.[2] Pristine **NU-1000** was obtained via treating the as-synthesized material (including removal of ligated and free benzoate/benzoic acid) via heating the isolated solid (~50 mg) in DMF (12 mL) with 0.5 mL of 8 M HCl (aq) at 100 °C oven for 18-24 h,[2-3] the solid was isolated, washed with DMF and acetone, and finally dried in a vacuum (~100 torr) oven for 30 min at 50 °C. **SALI-BA**, featuring four benzoates per Zr_6-oxo node, is the as-synthesized form of **NU-1000**.

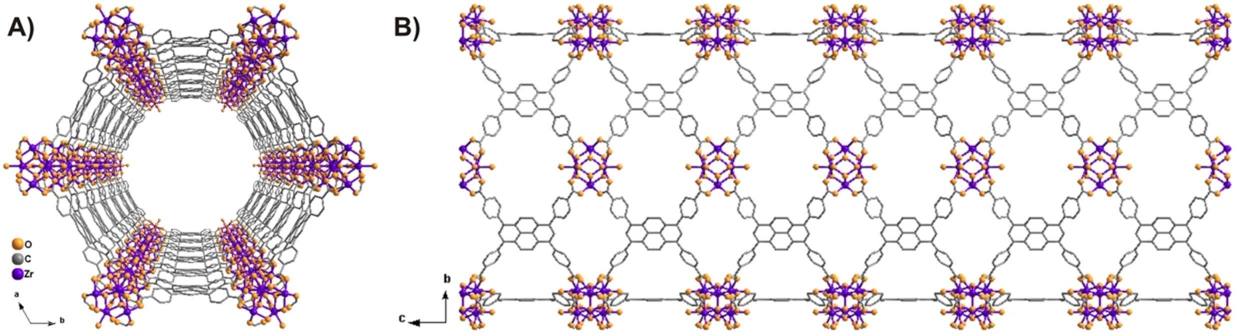

Figure SI-1. (a) Molecular rendering of **NU-1000** mesopore along the channel direction, and (b) side view highlighting the aperture of the triangular micropores (H-atoms were removed for clarity).

S2. Instrumentation

^{1}H NMR spectra of the SALI-CFG materials were recorded on a Agilent 400 MHz instrument after digesting the samples in 10% D_2SO_4/DMSO-d_6 and referenced to the residual solvent peak. Powder X-ray diffraction (PXRD) patterns were recorded on a Rigaku ATXG diffractometer equipped with an 18 kW Cu rotating anode, MLO monochromator, and a high-count-rate scintillation detector (in 0.05° step width with a 2 deg/min scanning speed). Diffuse reflectance infrared spectra (DRIFTS) were recorded on a Nicolet 7600 FTIR spectrometer equipped with an MCT detector. The spectra were collected in a KBr mixture under N_2 purge (samples were prepared under air); KBr was utilized as the background. Nitrogen isotherms were measured on a Micromeritics TriStar II 3020 at 77 K; for BET surface area analyses we ensured that the two consistency criteria described by Rouquerol et al.[4] and Walton et al.[5] were satisfied. Pore size distribution was calculated using Barrett-Joyner-Halenda (BJH) method with Halsey thickness curve and Kruk-Jaroniec-Sayari correction applied. Thermogravimetric analysis (TGA) was performed on a Mettler Toledo TGA under N2 flow and heated from room temperature to 700 °C (at 10 °C/min). Water vapor adsorption isotherms were collected on Hiden Isochema Intelligent Gravimetric Analyzer (IGA-200) equipped with water bath at 298 K; All isotherms were recorded using IGASwin software (v.1) that utilizes a linear driving force model (@ >96% equilibration had been reached) and corrects all data point for the buoyancy effects; thus the adsorbed mass at a given relative humidity point was the difference of mass gain at that point relative to the mass recorded in dry N_2 flow. Dry N_2 was used as carrier gas and was mixed with water vapor saturated N_2 gas stream, generated by

bubbling N_2 gas through a water reservoir at 298 K, at the required concentration or relative humidity; the total flow rate was 300 cc/min.

S3. Synthesis and characterization of SALI-derived materials

Preparation and characterization of **SALI-5** and **SALI-9** were described in our previous publication.[3b] Briefly, a 45 mg portion of activated pristine **NU-1000** (0.021 mmol) was taken in a 10 mL vial (VWR). Subsequently 3 mL of a 0.07 M solution of carboxylate functional group (CFG; e.g. decanoic acid 0.21 mmol) in a DMF solvent was added to the reaction vial, which was then capped and heated at 70 ^{0}C for 18-24 h with occasional swirling. The supernatant of the reaction mixture was decanted and the MOF sample was soaked in fresh hot DMF, filtered, washed sequentially with acetone and dichloromethane (40 and 30 mL each), and finally dried in a vacuum oven (~100 torr) for 30 min at 50 °C.

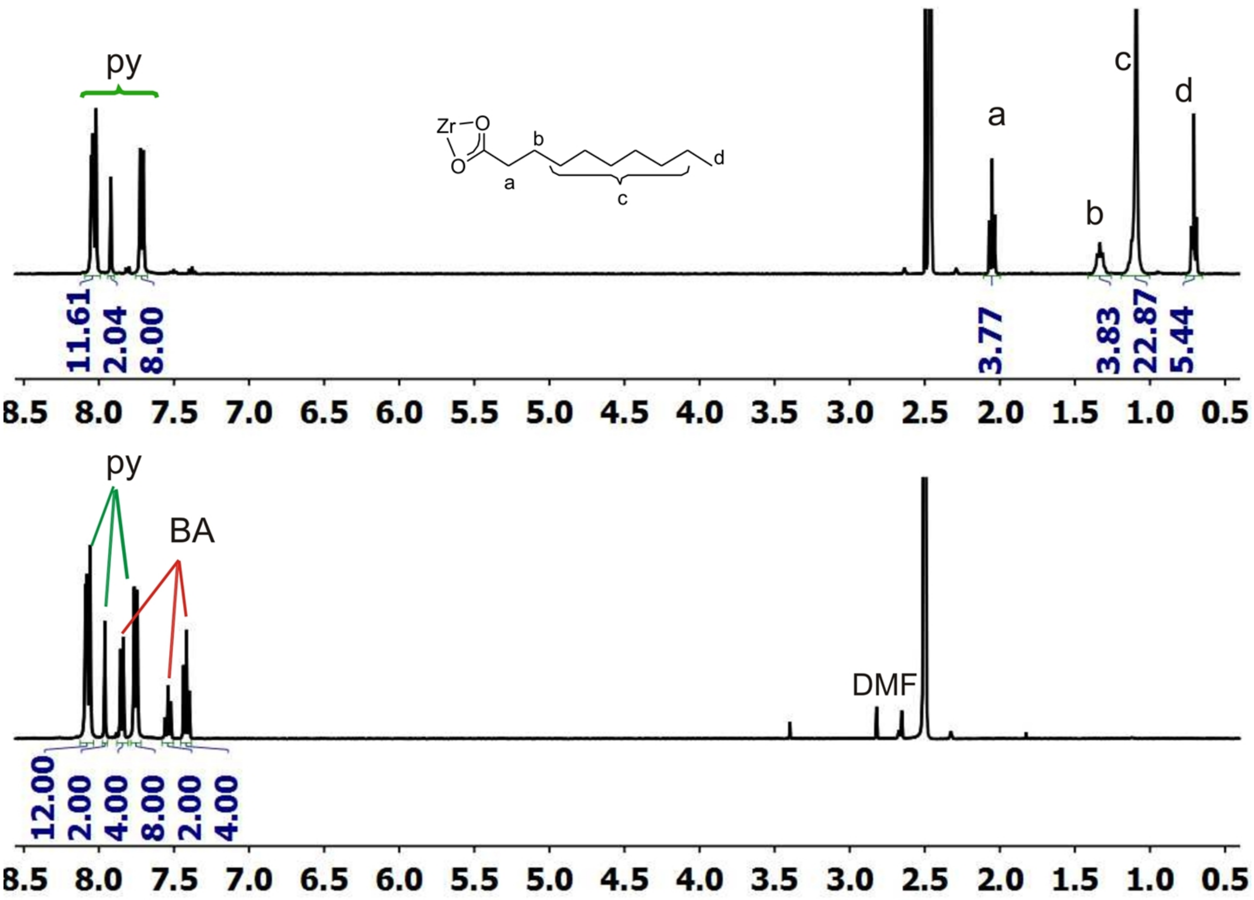

Figure SI-2. ^{1}H NMR spectra of **SALI-9'** (top) and **SALI-BA** (bottom) with characteristic peaks labeled. The spectra were recorded after digesting in 10% D_2SO_4/DMSO-d_6.

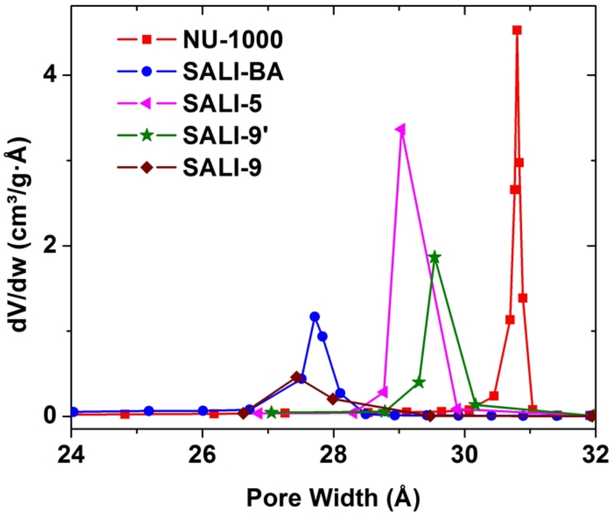

Figure SI-3. BJH pore size distribution calculated from desorption branch of the N_2 isotherms for **NU-1000**, **SALI-n**, and **SALI-n’** samples.

S4. Multi-cycle water vapor isotherms

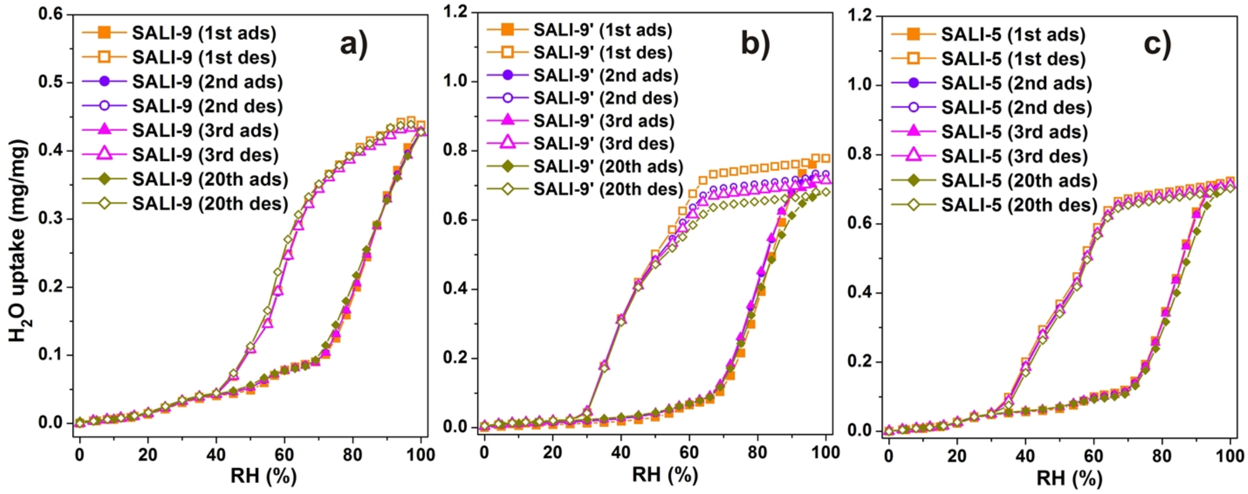

Figure SI-4. Multi-cycle water vapor isotherms of (a) **SALI-9**, (b) **SALI-9’**, and (c) **SALI-5** (adsorption: solid and desorption: open symbols).

S5. Characterization data for **NU-1000** and **SALI**-derived materials before and after multi-cycle water isotherms.

S5A. DRIFTS data

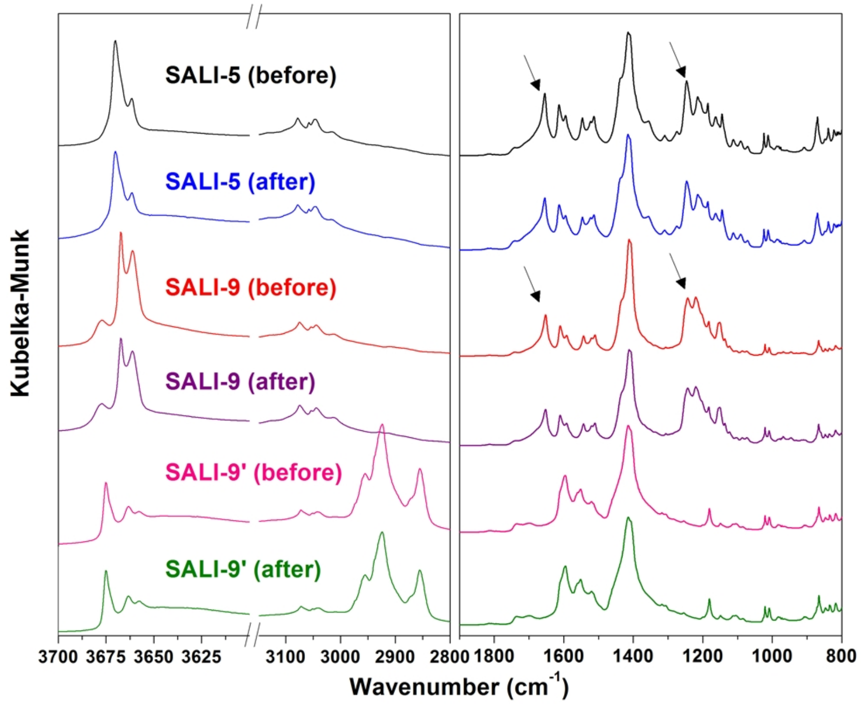

Figure SI-5. DRIFTS data of SALI-derived materials before and after recording the cycle of twenty water isotherms. The arrows (right panel) denote the signature for the perfluoroalkanes.

S5B. PXRD

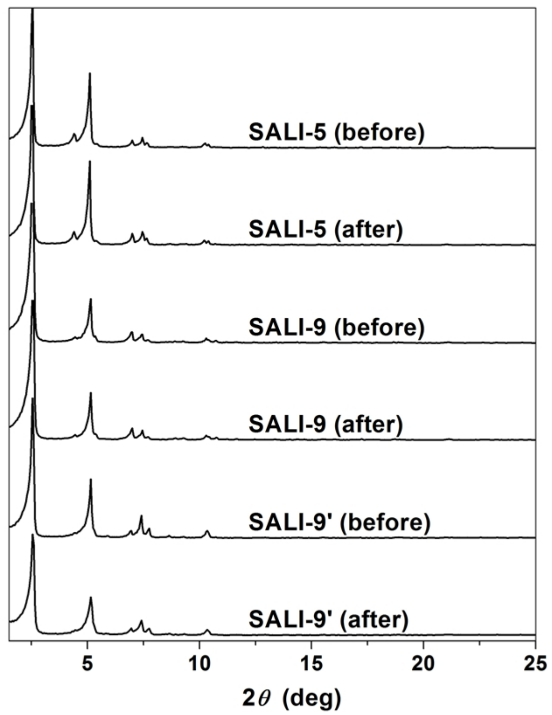

Figure SI-6. PXRD patterns of **SALI-CFG** before and after recording the cycle of twenty water isotherms.

S6 Water contact angle measurement

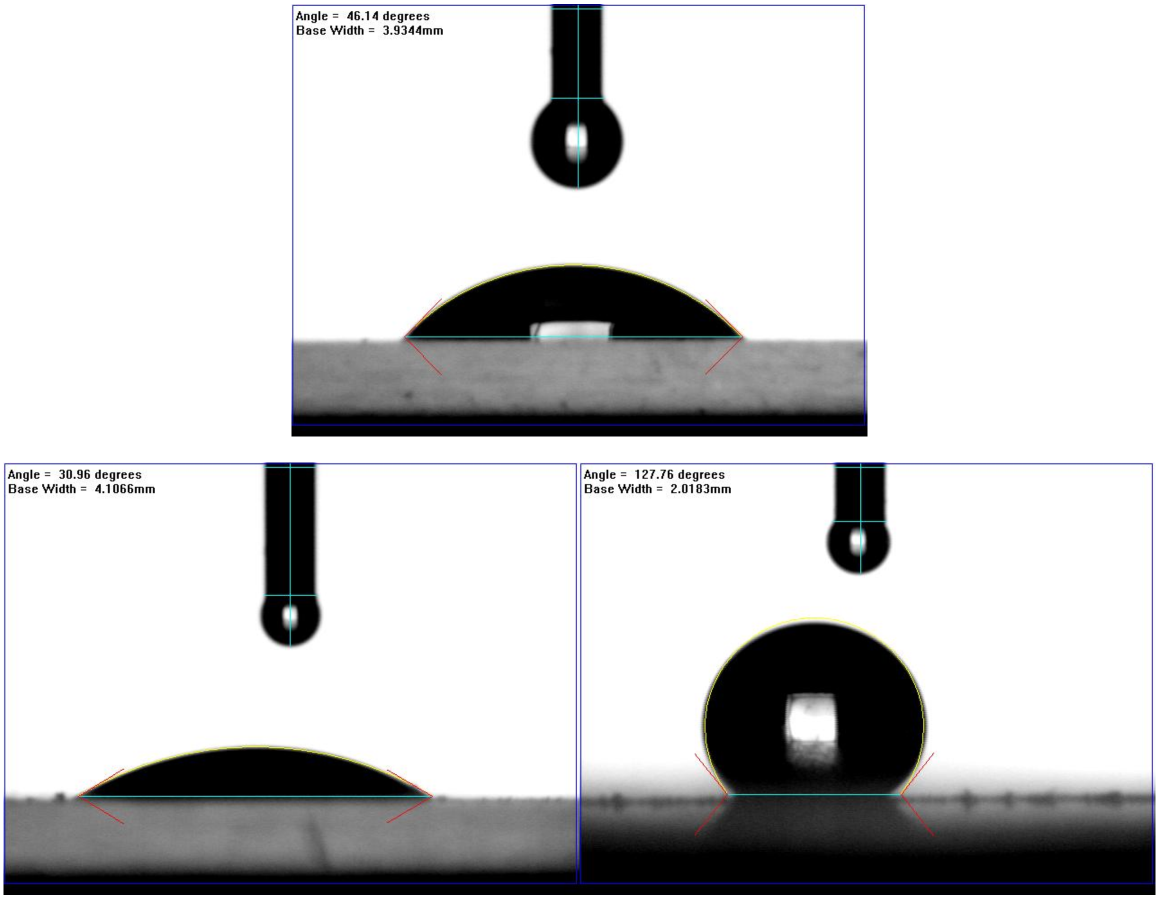

Figure SI-7. Water contact angle measurement of bare glass surface (top), pristine **NU-1000** smeared glass surface (bottom left), and **SALI-9** smeared glass surface (bottom right) highlighting bulk-phase wettability of the corresponding powder-packed surfaces. These data, however, are not conclusive for determination of the relative hydrophobicity of the MOF interior surface.

The water vapor isotherms for NU-1000 and SALI-9 suggest that in these mesoporous MOFs, it is difficult to define relative hydrophobicity within the MOF surface simply by the difference in the relative humidity where the corresponding capillary condensation takes place. While the contact angle measurements (**Figure SI-7**), a bulk measurement, indicate an enhanced hydrophobicity for **SALI-9**, the difference in corresponding vapor sorptions is mainly seen in total vapor uptake: for the **SALI-9** sample, a ~60% lower water vapor uptake relative to the pristine **NU-1000** was recorded (**Figure 4**).

S7 Details of the molecular dynamics (MD) simulation

S7A. Parameters for MD simulation

Molecular dynamics (MD) simulations were performed for water in three crystal structures: **NU-1000**, **SALI-BA**, and **SALI-5**. Atomic coordinates of the **NU-1000** framework atoms were obtained from previous work,[2] with the modification to the protonation state of the zirconium

nodes that has been recently determined from electronic structure calculations by Planas et al.[6] The final structure has four aqua and four hydroxyl ligands per zirconium node, which corresponds to the NU-1000-MIX-S12 structure in the paper by Planas et al.[6] To create a crystal of **SALI-BA**, four aqua and four hydroxyl ligands were removed from the **NU-1000** structure and replaced with four benzoates. Likewise, the **SALI-5** structure was created by replacing aqua and hydroxyl ligands with four perfluoroalkyl-carboxylate groups. The Materials Studio Forcite module[7] was used to optimize the structures of **SALI-BA** and **SALI-5** using the universal force field (UFF)[8] while fixing the lattice constant of the unit cell.

Non-bonded interactions between framework and water atoms, as well as water-water interactions, were modeled using the Lennard-Jones (LJ) 12-6 plus Coulomb potential:

$$V_{ij} = 4\varepsilon_{ij}\left[\left(\frac{\sigma_{ij}}{r_{ij}}\right)^{12} - \left(\frac{\sigma_{ij}}{r_{ij}}\right)^{6}\right] + \frac{q_i q_j}{4\pi\varepsilon_0 r_{ij}} \qquad \text{Eq. S1}$$

Subscripts i and j index the interacting atoms, r_{ij} is the distance between atoms i and j, ε and σ are the LJ parameters, q_i and q_j are the partial charges of the interacting atoms, and ε_0 is the dielectric constant. LJ parameters between atoms of different types were calculated using Jorgensen mixing rules:

$$\sigma_{ij} = \sqrt{\sigma_i \sigma_j} \qquad \text{Eq. S2}$$

$$\varepsilon_{ij} = \sqrt{\varepsilon_i \varepsilon_j} \qquad \text{Eq. S3}$$

All LJ interaction potentials were cut-off at 12.8 Å. Electrostatic interactions were summed using the Particle Mesh Ewald (PME) method as implemented in GROMACS (version 5.0.4)[9]. For **SALI-5**, perfluoroalkane chains were assigned bonded interaction potentials from OPLS[10] and allowed to move during the MD simulations. The partial charges on the perfluoroalkane chains were obtained from OPLS and the partial charges on the framework atoms were obtained from the EQeq method.[11] The LJ interaction parameters used in the simulations are listed in Table S1.

Table S1. Lennard-Jones (LJ) parameters used in the work.

Atom type	Mass (amu)	σ (nm)	ε (kJ/mol)
Zr	91.224	0.2783	0.2889
C	12.0107	0.3431	0.4396
O	15.9994	0.3118	0.2512
H	1.0079	0.2571	0.1842
F	18.9984	0.3000	0.2091

Molecular dynamics (MD) simulations were carried out for each MOF structure (**NU-1000**, **SALI-BA**, and **SALI-5**) using the GROMACS molecular simulation package (version 5.0.4). A leap-frog integrator was used in integrating Newton's equations of motion with a 0.0005 ps (0.5

fs) time step. The Verlet cutoff scheme was used to keep the neighborlist of each atom and the neighborlist was updated every 5 ps. Periodic boundary conditions were implemented in all directions.

A series of MD simulations were performed for each structure: 1) the simulation box was loaded with a saturation loading of the equilibrated TIP4P[12] water molecules at 300 K using the GROMACS solvation module, 2) a 100 ps NVT (constant number of molecules, volume, and temperature) simulation was performed without electrostatic interactions between water molecules, and 3) a 1 ns NVT simulation was carried out with full interaction potentials. For the solvation module, solvent molecules were removed from the simulation box if the distance between any atom of the framework and any atom of a TIP4P molecule was less than the sum of the van der Waals radii of the two atoms. The van der Waals radii were taken from Bondi.[13] The saturation loading of each structure varies because different ligands present in the structure take up different amounts of space in the simulation cell. Table S2 summarizes the saturation loadings obtained using the GROMACS solvation module and used in the subsequent MD simulations and the saturation loadings obtained from the experiments.

The temperature of the simulation box was maintained at 300 K for all simulations. The velocity re-scaling thermostat as implemented in GROMACS was used for the 100 ps NVT simulations. The Nose-Hoover thermostat was used for the subsequent 1 ns NVT simulations with a coupling constant of 0.1 ps.

Table S2. Saturation Loading of Water Molecules Used in the Simulations and from Experiments.

System	Unit Cell Volume (Å^3)	Total Number of H_2O in the Simulation Cell (N_{H2O})	Simulated H_2O Saturation Loading (mg/mg)	Experimental 1st cycle H_2O Saturation Loading (mg/mg)
**NU-1000**	45913.4	1123	1.35	1.14
**SALI-BA**	45913.4	1039	1.09	1.00
**SALI-5**	45913.4	995	0.96	0.72

S7B. Analysis of MD simulation results

Molecular snapshots were analyzed every 1 ps of the last 100 ps of the 1 ns NVT simulation of each structure. The water molecules in the hexagonal channels were extracted from each snapshot, and the average number of water molecules in the hexagonal channels was computed for each structure. Given that the N_2 accessible pore volumes, due to variation of the node functionalities, in these structure are different, analysis of the water density in the hexagonal channel is more straightforward to compare than simply comparing the number of water molecules. To calculate the water (number) density, the free volume of the hexagonal channel for each structure was computed based on the van der Waals radii from the UFF parameters,

subtracting the space occupied by the protruding ligands in the hexagonal channels. The average density of water in hexagonal channel is defined as:

$$\rho = \frac{N_{H2O}}{V_{Free\ volume}} \qquad \text{Eq. S4}$$

Results are given in Table S3. In addition, analyses were performed to analyze the number of water molecules near the Zr_6-node by placing a sphere with 15 Å diameter at the center of each Zr_6 node. Results are reported in Table S4.

Table S3. Average Number Density of Water Molecules in the Hexagonal Channels Obtained from the Last 100 ps of the 1 ns Simulation.

System	Average Number of H_2O Molecules (N_{H2O})	Free Volume (Å^3)	Average Number Density of H_2O (N/Å^3)
**NU-1000**	860.9 ± 4.3	23822	0.0361
**SALI-BA**	758.1 ± 3.4	22462	0.0337
**SALI-5**	731.5 ± 3.0	21742	0.0336

Table S4. Average Number of Water Molecules within a Sphere of 15 Å Diameter around the Zr_6 Nodes.

System	Average Number of H_2O Molecules (N_{H2O})
NU-1000	62.9
SALI-BA	53.1
SALI-5	50.3

References

(1) Stylianou, K. C.; Heck, R.; Chong, S. Y.; Bacsa, J.; Jones, J. T. A.; Khimyak, Y. Z.; Bradshaw, D.; Rosseinsky, M. J. *J. Am. Chem. Soc.* **2010**, *132*, 4119.

(2) Mondloch, J. E.; Bury, W.; Fairen-Jimenez, D.; Kwon, S.; DeMarco, E. J.; Weston, M. H.; Sarjeant, A. A.; Nguyen, S. T.; Stair, P. C.; Snurr, R. Q.; Farha, O. K.; Hupp, J. T. *J. Am. Chem. Soc.* **2013**, *135*, 10294.

(3) (a) Deria, P.; Bury, W.; Hupp, J. T.; Farha, O. K. *Chem. Commun.* **2014**, *50*, 1965; (b) Deria, P.; Mondloch, J. E.; Tylianakis, E.; Ghosh, P.; Bury, W.; Snurr, R. Q.; Hupp, J. T.; Farha, O. K. *J. Am. Chem. Soc.* **2013**, *135*, 16801.

(4) Rouquerol, J.; Llewellyn, P.; Rouquerol, F. *Stud. Surf. Sci. Catal.* **2007**, *160*, 49.

(5) Walton, K. S.; Snurr, R. Q. *J. Am. Chem. Soc.* **2007**, *129*, 8552.

(6) Planas, N.; Mondloch, J. E.; Tussupbayev, S.; Borycz, J.; Gagliardi, L.; Hupp, J. T.; Farha, O. K.; Cramer, C. J. *J. Phys Chem. Lett.* **2014**, *5*, 3716.
(7) Accelrys Software Inc: San Diego, CA 92121, USA.
(8) Rappé, A. K.; Casewit, C. J.; Colwell, K. S.; Goddard, W. A.; Skiff, W. M. *J. Am. Chem. Soc.* **1992**, *114*, 10024.
(9) Pronk, S.; Pall, S.; Schulz, R.; Larsson, P.; Bjelkmar, P.; Apostolov, R.; Shirts, M. R.; Smith, J. C.; Kasson, P. M.; van der Spoel, D.; Hess, B.; Lindahl, E. *Bioinformatics* **2013**, *29*, 845.
(10) Watkins, K.; Jorgensen , W. L. *J. Phys. Chem. A* **2001**, *105*, 4118.
(11) Wilmer, C. E.; Kim, K. C.; Snurr, R. Q. *J. Phys. Chem. Lett.* **2012**, *3*, 2506.
(12) Jorgensen, W. L.; Chandrasekhar, J.; Madura, J. D.; Impey, R. W.; Klein, M. L. *J. Chem. Phys.* **1983**, *79*, 926.
(13) Bondi, A. *J. Phys. Chem.* **1964**, *68*, 441.
